# SLC5A3 is important for cervical cancer cell growth

**DOI:** 10.7150/ijbs.84570

**Published:** 2023-05-27

**Authors:** Li Li, Fang-rong Shen, Qunxian Cheng, Jing Sun, Hang Li, Hua-ting Sun, Xia Cai, Mengting Chen, Baohua Yang, Lifeng Wang, Ling Xu

**Affiliations:** 1 Department of Obstetrics and Gynecology, Minhang Hospital, Fudan University, Shanghai, China.; 2 Obstetrics and Gynecology Department, The First Affiliated Hospital of Soochow University, Suzhou, China.

## Abstract

Novel molecular targets for cervical cancer must be identified. This study examined the role of SLC5A3, a myo-inositol transporter, in the pathogenesis of cervical cancer. Through boinformatics analysis, we showed that the *SLC5A3* mRNA levels were upregulated in cervical cancer tissues. The upregulated *SLC5A3* mRNA levels were negatively correlated with survival and progression-free interval. Genes co-expressed with *SLC5A3* were enriched in multiple signaling cascades involved in cancer progression. In primary/established cervical cancer cells, *SLC5A3* shRNA/knockout (KO) exerted growth-inhibitory effects and promoted cell death/apoptosis. Furthermore, *SLC5A3* knockdown or KO downregulated myo-inositol levels, induced oxidative injury, and decreased Akt-mTOR activation in cervical cancer cells. In contrast, supplementation of myo-inositol or n-acetyl-L-cysteine or transduction of a constitutively active Akt1 construct mitigated *SLC5A3* KO-induced cytotoxicity in cervical cancer cells. Lentiviral SLC5A3 overexpression construct transduction upregulated the cellular myo-inositol level and promoted Akt-mTOR activation, enhancing cervical cancer cell proliferation and migration. The binding of TonEBP to the *SLC5A3* promoter was upregulated in cervical cancer. *In vivo* studies showed that intratumoral injection of SLC5A3 shRNA-expressing virus arrested cervical cancer xenograft growth in mice. SLC5A3 KO also inhibited pCCa-1 cervical cancer xenograft growth. The SLC5A3-depleted xenograft tissues exhibited myo-inositol downregulation, Akt-mTOR inactivation, and oxidative injury. Transduction of sh-TonEBP AAV construct downregulated SLC5A3 expression and inhibited pCCa-1 cervical cancer xenograft growth. Together, overexpressed SLC5A3 promotes growth of cervical cancer cells, representing as a novel therapeutic oncotarget for the devastating disease.

## Introduction

Globally, cervical cancer is estimated to be associated with more than 320,000 mortalities every year 1, 2. The incidence (13.1%) and mortality (6.1%) rates of cervical cancer in women are the fourth highest among cancers 3, 4.

Currently, a curative therapeutic strategy is not available for patients with advanced, recurrent, or metastatic cervical cancer 3-5. The efficacy of current treatments, including traditional cisplatin/paclitaxel chemotherapy, bevacizumab targeted therapy 6, 7, and immunotherapy (immune checkpoint inhibitors) 8, is < 50% in these patients 4, 9. Thus, there is a need to identify novel signaling targets and develop corresponding targeted therapies for advanced, recurrent, and metastatic cervical cancer 6, 10.

*SLC5A3*, which encodes a Na^+^/*myo-*inositol (MI) cotransporter, is located at q22 on chromosome 21 and contains one promoter and two exons spanning 2157 nucleotides 11, 12. The SLC5A3 protein comprises 718 amino acid residues and is expressed in different human tissues 12. The expression of SLC5A3 is reported to be upregulated in patients with Down's syndrome 12-14. Andronic *et al.* demonstrated that hypotonic stress upregulated SLC5A3 expression and MI transport in HEK293 cells, suggesting that SLC5A3 regulates mammalian cell hypotonic volume 15.

Some studies have examined the function of SLC5A3 in the pathogenesis of human cancer. For example, SLC5A3-dependent MI transport, which promotes nutrient dependency, is required for acute myeloid leukemia (AML) cell proliferation 16. *SLC5A3* silencing decreased MI contents and arrested AML cell proliferation 16. This study demonstrated that SLC5A3 overexpression promoted cervical cancer cell growth.

## Materials and methods

### Reagents

CCK-8, puromycin, cell culture medium, serum, MI, N-Acetyl-L-cysteine (NAC) as well as RNA reagents, fluorescence dyes, antibodies and the caspase inhibitors were provided by Dr. Cui 17 or purchased from Sigma (St. Louis, Mo).

### Cells

“pCCa-1”, “pCCa-2”, “pCCa-3” primary cancer cells (derived from three patients), the primary human cervical epithelial cells (“HCerEpC1”), HeLa immortalized cells and Ect1/E6E7 epithelial cells were from Dr. Cao 18. Cells were cultivated under the described high glucose (4.5g/L glucose) medium 19. The protocols of this study were reviewed by the Ethics Committee of Minhang Hospital, Fudan University (Shanghai, China).

### Human tissues

The cervical cancer tissues along with the matched adjacent normal cervical epithelial tissues were from twenty (20) cervical cancer patients (written-informed consent, administrated at authors' institution). The immunohistochemistry (IHC) staining in four μm-thick tissue slides were reported previously 20.

### shRNA or overexpression

The sequences encoding three different shRNAs targeting human *SLC5A3*, shSLC5A3 (seq1, targeting: GCACTTACACTTATGATTATT), shSLC5A3 (seq2, targeting: GACTAATTCTGAAGCATATTT) and shSLC5A3 (seq3, targeting: ACCCTAACCATTGGCATATAT), or the SLC5A3 cDNA sequence (hSLC5A3[NM_006933.7]), were individually inserted into GV248 lentiviral construct (Genechem). Each of the construct was co-transfected to HEK-293 cells along with lentivirus envelope constructs (Genechem). The generated lentiviral particles were added to cultured cervical cancer cells or epithelial cells at MOI=11.6 for 48h. Afterwards, puromycin was added for another 96h to select stable cells. For animal xenograft studies, the two SLC5A3 shRNA (-seq1/3) sequence or the scramble control shRNA sequence (shC) was inserted into an AAV9 construct (Genechem). The shRNA AAV was then generated. Silencing or overexpression (hSLC5A3[NM_006933.7]) of TonEBP (tonicity-responsive enhancer-binding protein) was through exact the same procedure *in vitro* and *in vivo*. Two different shRNAs against TonEBP, shTonEBP-seq1 (targeting: GCAGCAGATTTCATCAAATAT) and shTonEBP-seq2 (targeting: GCAGAGTAACTGGACGAAATA), were utilized. Parental cells (“Pare”) were always utilized as the control cells.

### CRISPR/Cas9

Cells were transfected with lentiviral particles with Cas9-expressing construct and stable cells were formed following selection. The two different sequences encoding small-guide (sgRNA) targeting SLC5A3 were each inserted into a lenti-CRISPR/Cas9-KO-puro construct (Genechem). sg-1 targets ATCCCAATTTACATCCGGTC with AGG PAM sequence. sg-2 targets TCCCAATTTACATCCGGTCA with GGG PAM sequence. The construct was transfected to HEK-293 cells to generate lentiviral particles. The particles were then added to Cas9 stable cells, and puromycin added to select stable colonies. *SLC5A3* KO screening was carried out.

### Immunofluorescence and ChIP assays

In the cell culture plate, the glass slide was first fixed and washed with PBS for three times. Next, the 0.5% Triton X-100 was added for permeabilization. Different fluoresce dyes were added to the glass slide and the redundant fluoresce dyes washed off four times with PBS. Water absorbent paper was utilized to suck up the liquid and the fluoresce signalings were observed and collected under a fluorescence microscope (Zeiss). TonEBP chromosome immunoprecipitation (ChIP)'s protocols were described previously 21.

### Myo-inositol (MI) detection and superoxide dismutase (SOD) activity

In brief, MI contents were detected through a MI assay kit (ab252896, abcam, Shanghai, China). The SOD activity in fresh tumor tissues was measured under the SOD ELISA kit (Thermo-Fisher Invitrogen, Shanghai, China).

### Constitutively-active Akt1

The viral particles with the S473D constitutively-active Akt1 (caAkt1) were from Dr. Xu 22 and added to pCCa-1 primary cancer cells, with stable cells formed following selection 22.

### Other assays

CCK-8 viability assay, Trypan blue staining of cell death, Caspase-3 activity detection, “Transwell” assay of cell migration, nuclear EdU staining (quantifying cell proliferation), nuclear TUNEL staining of cell death, thiobarbituric acid reactive substance (TBAR) assay, CellROX staining assay of ROS (reactive oxygen species) production and mitochondrial depolarization (JC-1 staining) were reported in other studies 23. qRT-PCR and Western blotting protocols were reported early 17. mRNA primers for *SLC5A3* and *SLC5A11* were described early 24, 25. Figure **S1** included the uncropped blotting images.

### Xenograft study

The female nude mice, weighing 18.4-19.1g, were provided by Changzhou Cavens Experimental Animal Co (Changzhou, China). pCCa-1 cells in non-serum medium, at 5 × 10 ^6^ cells per mouse, were subcutaneously (*s.c.*) injected to the mice. The xenografts were formed afterwards. Mice were thereafter intratumorally injected with AAV-packed shRNAs (at 1.2 × 10 ^9^ PFU, 8.5 μL). The recordings of the xenografts and mice were reported previously 20.

### Statistical difference

All the *in vitro* cellular experiments were repeated five times (n = 5). The statistical methods were described early 23. ***P*** values < 0.05 indicated statistically significant. “N. S.” stands for non-statistical difference (***P*** > 0.05).

## Results

### SLC5A3 is upregulated in cervical cancer

**Figure 1:**
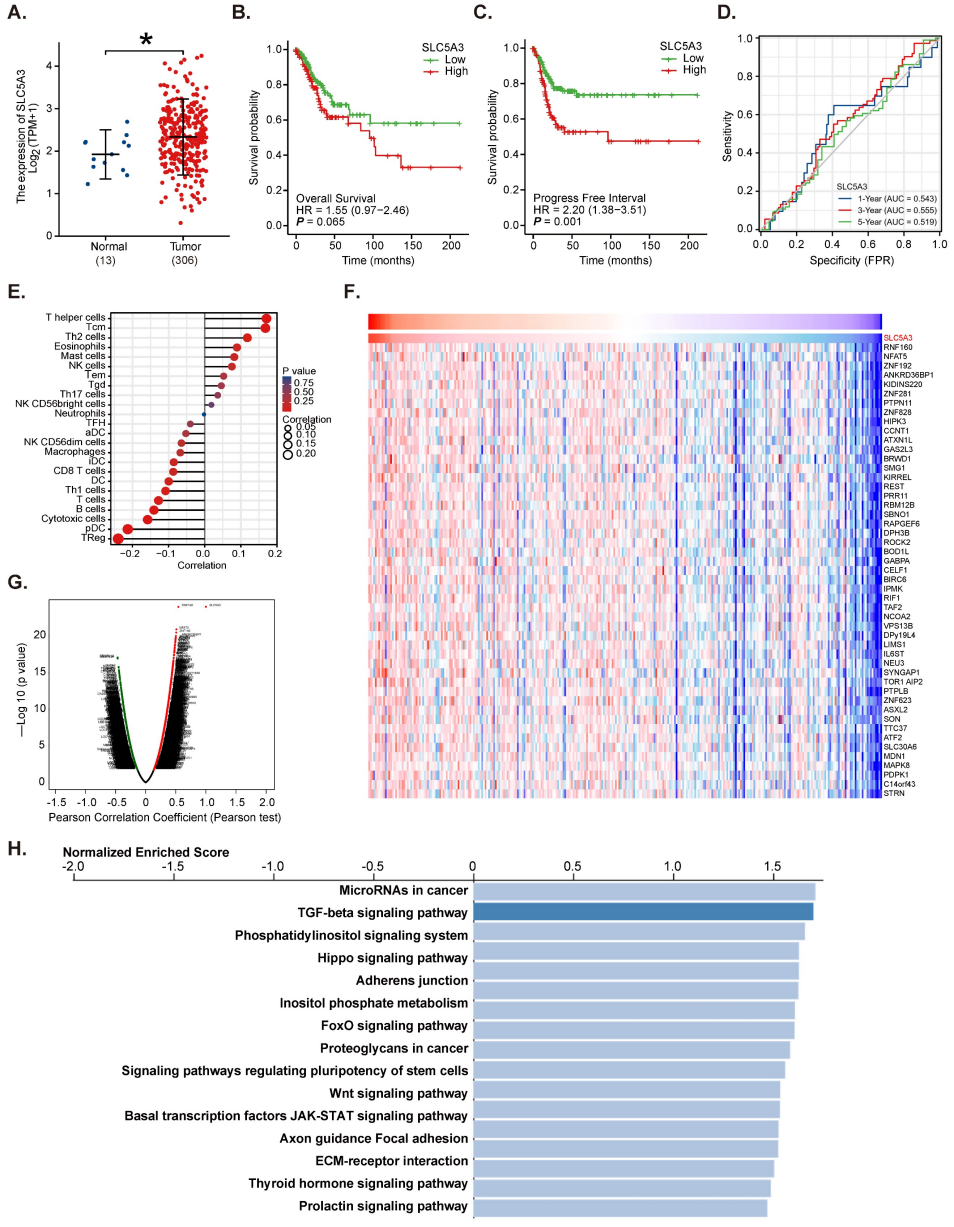
**SLC5A3 is upregulated in cervical cancer.** TCGA cervical cancer cohort combining Genotype-Tissue Expression (GTEx) program revealed *SLC5A3 mRNA* transcripts in cervical cancer tissues (“Tumor”, n = 306) and ten (10) normal tissues plus three (3) parecancer normal tissues (total 13, “Normal”) (**A**).The overall survival (**B**) and progress free interval (**C**) in *SLC5A3*-low and *SLC5A3*-high cervical cancer patients from TCGA cervical cancer cohort. *ROC* curve showed the potential value of SLC5A3 overexpression in predicting cervical cancer patients' survival (1-5 years) (**D**). The multiple omics and immune infiltration analysis of the association between the *SLC5A3* expression and immune cells infiltration (**E**). The heat map (**F**) and volcano map (**G**) of DEGs with *SLC5A3* in TCGA cervical cancer cohort were presented; KEGG of top 15 enriched pathways of *SLC5A3*-associated DEGs were presented (**H**).

*The Cancer Genome Atlas (TCGA)* database and Genotype-Tissue Expression (GTEx) dataset, which comprise RNA sequencing data of 10 healthy tissues, three paracancerous tissues, and 306 cervical cancer tissues, were analyzed. The *SLC5A3* mRNA expression levels in tumor tissues were markedly higher than those in paracancerous and healthy tissues (***P*** < 0.05) (Figure **1A**). Analysis of TCGA cervical cancer datasets revealed that patients with cervical cancer exhibiting SLC5A3 upregulation were associated with poor overall survival (***P*** = 0.065) (Figure **1B**) and progression-free interval (PFI) (***P*** = 0.001, Figure **1C**). The *receiver operating characteristic* curve analysis suggested that SLC5A3 upregulation can predict the 1-5 year survival rates of cervical cancer patients (Figure **1D**).

Immune cell infiltration analysis based on transcriptome and other omics data revealed that *SLC5A3* upregulation was correlated with the infiltration of different immune cells (Figure **1E**). The LinkedOmics functional model was used to examine genes co-expressed with *SLC5A3* in TCGA cervical cancer cohort. The top 50 significant genes positively correlated with SLC5A3 were retrieved (Figure **1F**-**G**). Kyoto Encyclopedia of Genes and Genomes analyses revealed that genes co-expressed with *SLC5A3* were enriched in multiple pathways involved in cancer progression, including Hippo, FOXO, JAK-STAT, and extracellular matrix pathways (Figure **1H**). Thus, bioinformatics analysis revealed that SLC5A3 is upregulated in cervical cancer.

### SLC5A3 is upregulated in clinical cervical cancer tissues and patient-derived or established cervical cancer cells

**Figure 2:**
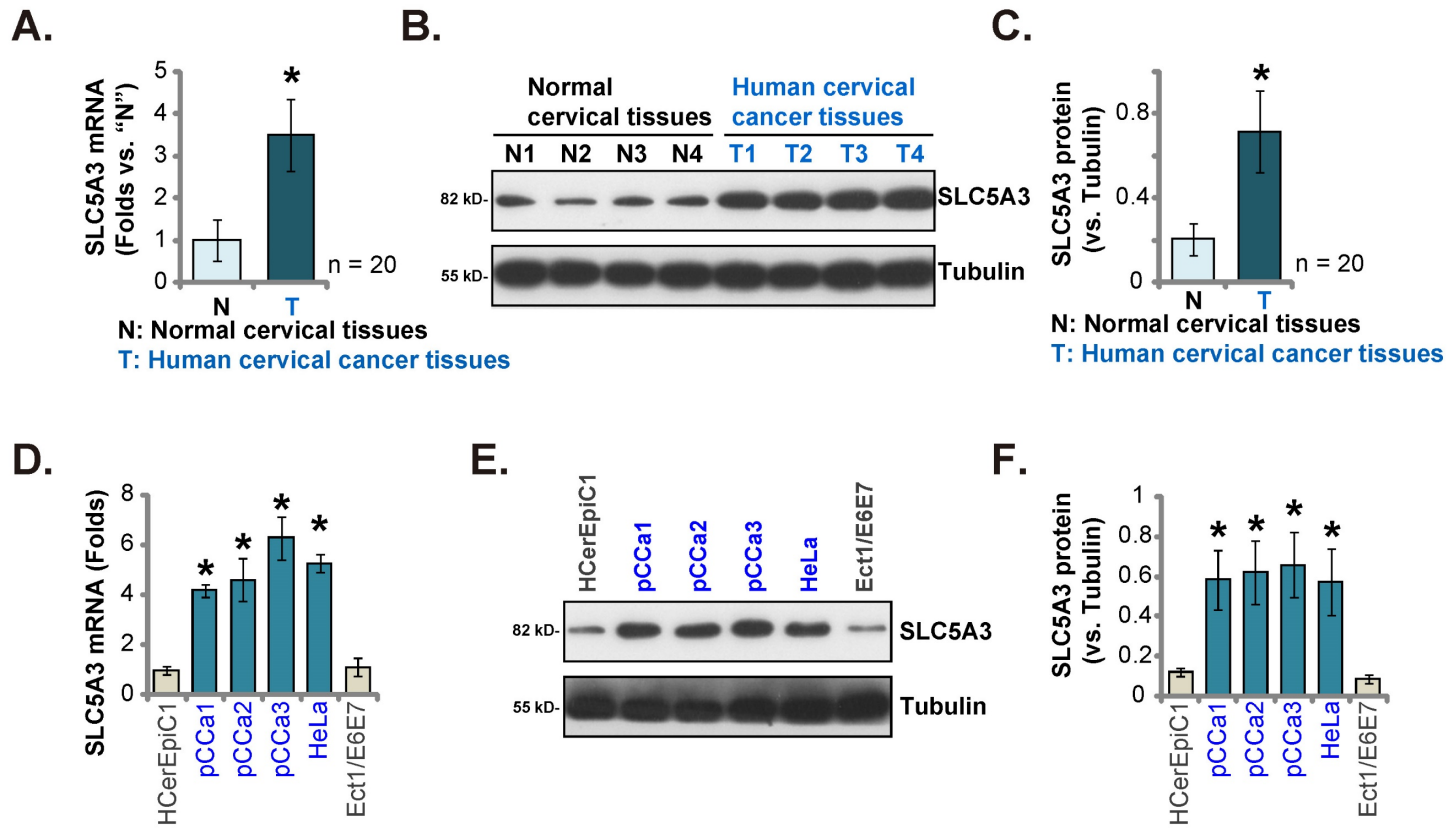
** SLC5A3 is upregulated in clinical cervical cancer tissues and patient-derived or established cervical cancer cells.** SLC5A3 expression in the described tissues of twenty (20) cervical cancer patients (n = 20) was tested (**A**-**C**). SLC5A3 expression in the described cells was also tested (**D-F**). *P < 0.05 versus “N” tissues/“HCerEpiC1” cells.

Next, the clinical specimens of cervical cancer (n = 20) were obtained. The *SLC5A3* mRNA levels in cancer tissues (“T”) were higher than those in healthy epithelial tissues (N”) (Figure **2A**). The protein levels of SLC5A3 were upregulated in the cancer tissue lysates prepared from four representative clinical specimens (Figure **2B**). Western blotting analysis results of all 20 sets of tissue lysates were combined, which revealed significant SLC5A3 protein upregulation in cancer tissues (Figure **2C**). *SLC5A3* mRNA and protein expression was significantly upregulated in the primary human cervical cancer cells (pCCa-1/-2/-3 cells, which were obtained from Dr. Cao 18) and the immortalized HeLa cells (Figure **2D**-**F**), but were low in the cervical epithelial cells (HCerEpC1 18 and Ect1/E6E7 cells) (Figure **2D**-**F**).

### Silence of *SLC5A3* produces anti-cervical cancer cell activity

**Figure 3:**
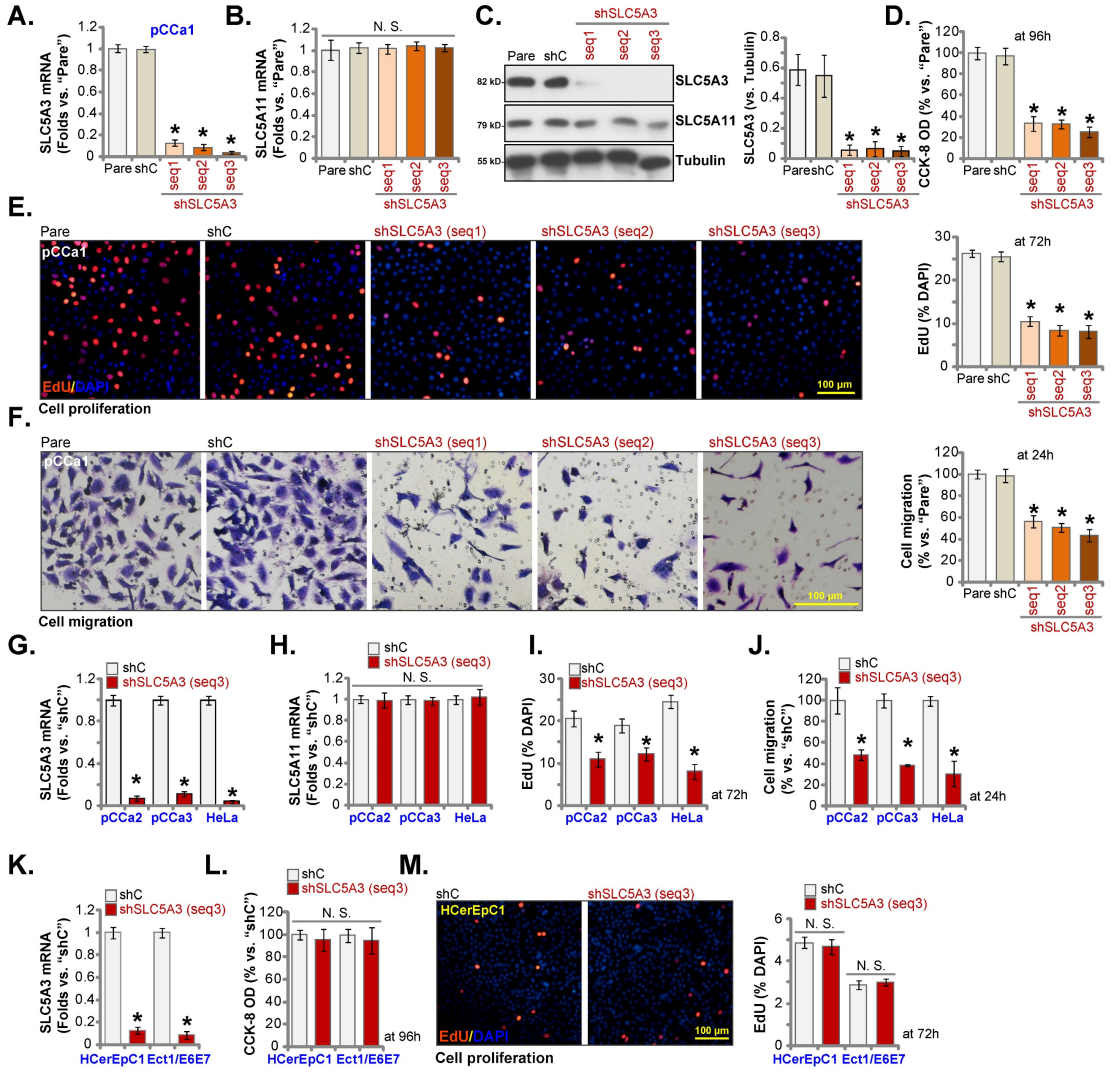
** Silence of SLC5A3 produces anti-cervical cancer cell activity.** pCCa-1 primary cells expressing the applied lentiviral SLC5A3 shRNA [shSLC5A3 (seq1/2/3)] or the control shRNA (shC) were established, with listed mRNAs and proteins tested (**A**-**C**). The above pCCa-1 cells were cultivated , cell viability (**D**), proliferation (**E**) and migration (**F**) were examined by the described methods. pCCa-2 and pCCa-3 primary cancer cells, HeLa cells, HCerEpC1 cells or Ect1/E6E7 epithelial cells were infected with lentiviral SLC5A3 shRNA (seq3) or shC, and stable cells formed. Listed mRNAs were shown (**G**, **H** and **K**). EdU nuclear incorporation (**I** and **M**), cell migration (**J**) and viability (**L**) were examined, and results quantified. * ***P*** < 0.05 *vs.* “shC” cells.

*SLC5A3* was knocked down using lentiviral particles encoding three different shRNAs targeting *SLC5A3*. sh-SLC5A3 (seq1/2/3) constructs were transduced into pCCa-1 primary cells. The infected cells were further selected using a puromycin-supplemented medium to obtain stable colonies. As shown in Figure **3A**, transduction with sh-SLC5A3 (seq1/2/3) constructs markedly downregulated the *SLC5A3* mRNA levels in pCCa-1 cells but did not affect the *SLC5A11* mRNA levels (Figure **3B**). Consistently, transduction with sh-SLC5A3 (seq1/2/3) constructs downregulated the SLC5A3 protein levels in pCCa-1 cells (Figure **3C**) but did not affect the SLC5A11 protein levels (Figure **3C**).

CCK-8 assay showed that *SLC5A3* knockdown decreased the viability of pCCa-1 cells (Figure **3D**). The number of 5-ethynyl-2′-deoxyuridine (EdU)-positive nuclei was markedly downregulated in *SLC5A3* knockdown pCCa-1 cells, suggesting that *SLC5A3* knockdown inhibited cell proliferation (Figure **3E**). Additionally, *SLC5A3* knockdown suppressed the *in vitro* migration of pCCa-1 primary cells (Figure **3F**).

Transduction with sh-SLC5A3 (seq3) lentiviral particles downregulated the expression of *SLC5A3* mRNA by more than 90% in pCCa-2/pCCa-3 primary cells or HeLa cells (Figure **3G**) but did not affect the expression of *SLC5A11* mRNA (Figure **3H**). *SLC5A3* knockdown markedly inhibited the proliferation (Figure **3I**) and *in vitro* migration of cancer cells (Figure **3J**). Similarly, transduction with sh-SLC5A3 (seq3) lentiviral particles downregulated the *SLC5A3* mRNA levels in HCerEpC1 and Ect1/E6E7 epithelial cells (Figure **3K**). However, *SLC5A3* knockdown did not significantly reduce the viability (Figure **3L**) and number of EdU-positive nuclei (Figure **3M**) in the epithelial cells.

### Apoptosis is induced in *SLC5A3* knockdown cervical cancer cells

**Figure 4:**
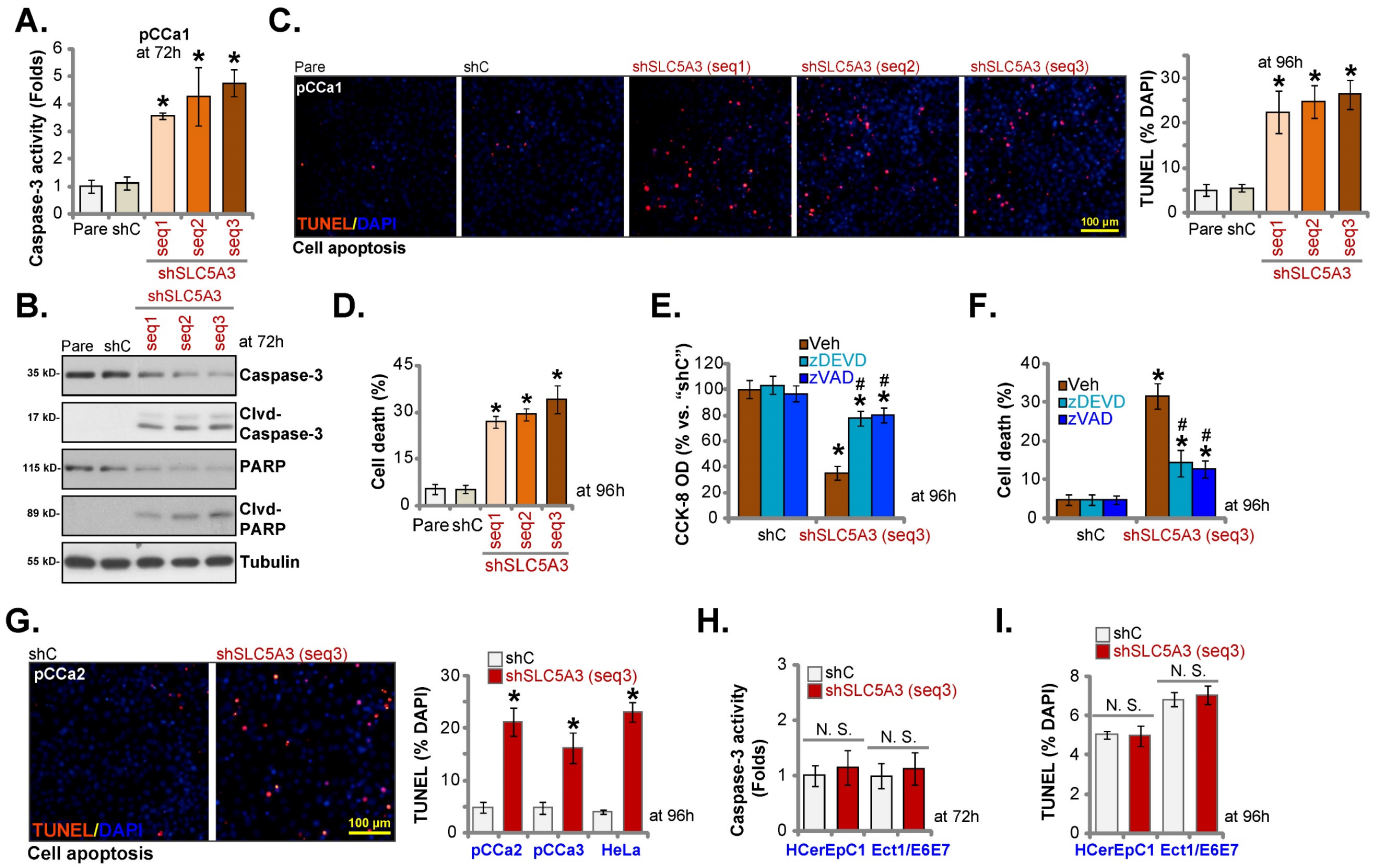
** Apoptosis is induced in *SLC5A3* knockdown cervical cancer cells.** The pCCa-1/2/3 primary cells (**A**-**D** and **G**), HeLa cells (**G**), HCerEpC1 cells or Ect1/E6E7 epithelial cells (**H** and **I**), with the described SLC5A3 shRNA or the control shRNA (shC), were maintained in complete medium for designated time, caspase-PARP activation was examined (**A**, **B**, **H**); Cell apoptosis was examined by TUNEL nuclear staining (**C, G** and **I**) assays, with results quantified. Cell death was measured by the Trypan blue assay (**D**). The shC- or shSLC5A3 (seq3)-expressing pCCa-1 cells were co-treated with 40 μM of z-VAD-fmk (40 μM) or z-DEVD-fmk for 96h, cell viability and cell death were measured and quantified by CCK-8 (**E**) and Trypan blue staining (**F**) assays. * ***P*** < 0.05 *vs.* “shC” cells. **^#^ *P*** < 0.05 vs. “Veh” treatment (**E** and **F**).

Cervical cancer cell growth arrest and proliferation inhibition can activate cell apoptosis 26-28. Thus, the effect of *SLC5A3* knockdown on cervical cancer cell apoptosis was examined. Transduction with sh-SLC5A3 (seq1/2/3) upregulated the CASP3 activity (Figure **4A**) and the levels of cleaved CASP3 and PARP1 in pCCa-1 cells (Figure **4B**). The number of TUNEL-positive nuclei was upregulated in *SLC5A3* knockdown pCCa-1 cells (Figure **4C**). Furthermore, *SLC5A3* knockdown significantly increased the number of trypan blue-positive pCCa-1 cells (Figure **4D**), indicating the upregulation of cell death. Treatment with caspase-apoptosis inhibitors, including z-DEVD-fmk and z-VAD-fmk, mitigated the *SLC5A3* knockdown-induced downregulation of cell viability (Figure **4E**) and upregulation of cell death (Figure **4F**) in pCCa-1 cells.

Transduction with sh-SLC5A3 (seq3) significantly induced apoptosis and increased the number of TUNEL-positive nuclei in pCCa-2 and pCCa-3 primary cervical cancer cells and HeLa cells (Figure **4G**) but did not promote CASP3 activation (Figure **4H**) or induce cell apoptosis (TUNEL assays, Figure **4I**) in HCerEpC1 and Ect1/E6E7 epithelial cells.

### *SLC5A3* knockout causes remarkable anti-cervical cancer activity

**Figure 5:**
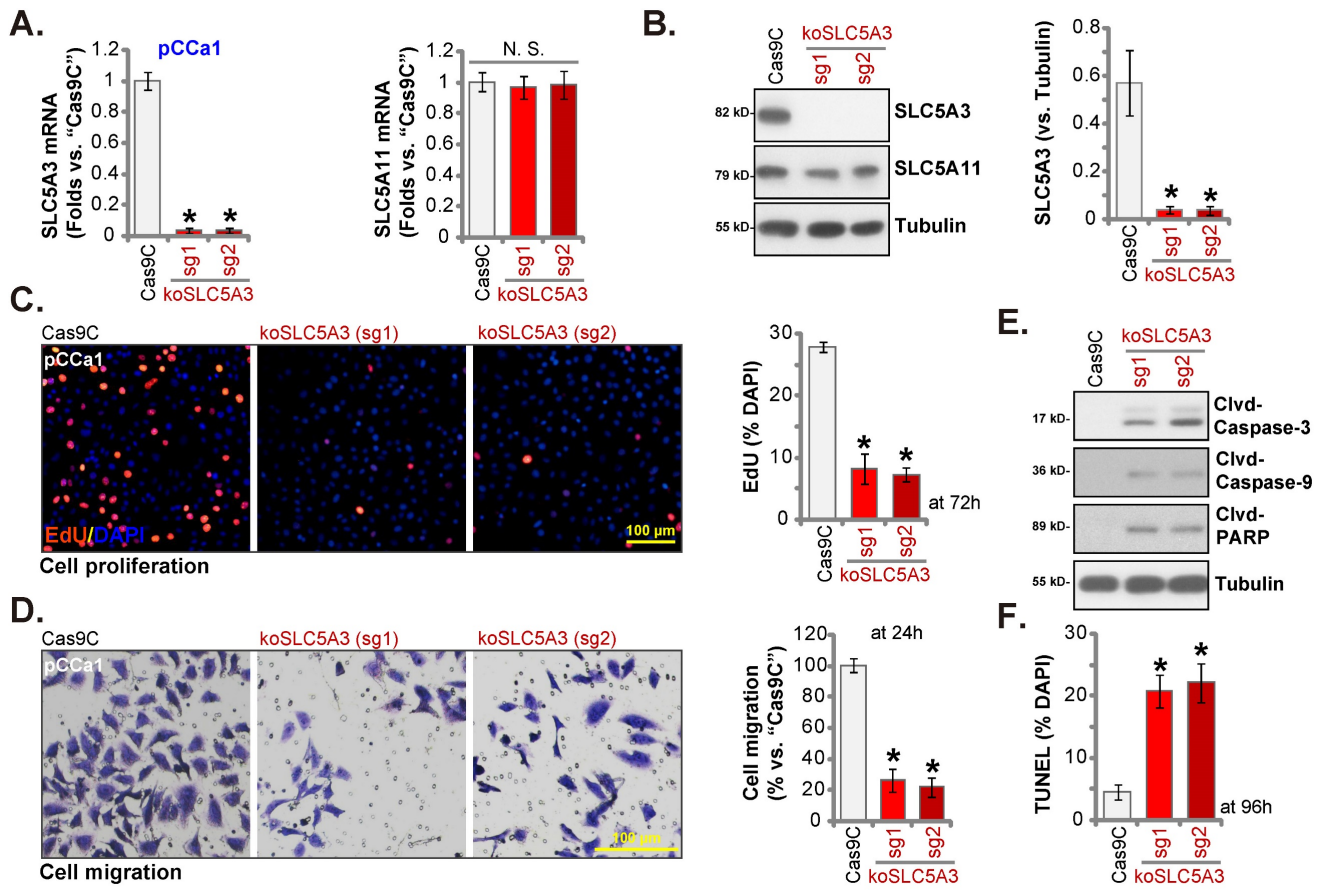
** SLC5A3 knockout causes remarkable anti-cervical cancer activity.** pCCa-1 cells, stably expressing the CRISPR/Cas9-SLC5A3-knockout (KO) construct encoded two different sgRNAs: koSLC5A3 (sg1) or koSLC5A3 (sg2), as well as the Cas9 control construct (“Cas9C”), were cultured, and listed mRNAs /proteins examined (**A** and **B**). The exact same number of above viable pCCa-1 cells were cultured for designated hours, and EdU nuclear incorporation (**C**), migration (**D**), caspase-3 activity (**E**) and TUNEL nuclear incorporation (**F**) were examined. * ***P*** < 0.05 *vs.* “Cas9C” cells.

SLC5A3 was knocked out using CRISPR/ Cas9 system. Two CRISPR/Cas9-SLC5A3 knockout (KO) constructs, which encoded different single-guide RNAs (sgRNAs) against *SLC5A3* [koSLC5A3 (sg1) and koSLC5A3 (sg2)], were individually transfected into Cas9-expressing pCCa-1 cells. After *SLC5A3* KO screening, stable SLC5A3 KO pCCa-1 primary cells (“koSLC5A3” cells) were established. The expression of SLC5A3 was depleted in koSLC5A3 cells (Figure **5A**-**B**) although SLC5A11 expression was unaltered (Figure **5A**-**B**). The percentage of EdU-positive nuclei was downregulated in koSLC5A3 pCCa-1 cells (Figure **5C**), suggesting the inhibition of cell proliferation. Furthermore, *SLC5A3* KO markedly suppressed pCCa-1 cell migration (Figure **5D**). The expression levels of cleaved CASP3, PARP1, and CASP9 (apoptotic marker proteins) were upregulated in koSLC5A3 pCCa-1 cells (Figure **5E**). Consistently, the percentage of TUNEL-positive nuclei was upregulated in koSLC5A3 pCCa-1 cells (Figure **5F**). Thus, *SLC5A3* KO exerted anti-cervical cancer effects.

### *SLC5A3* knockdown/KO promotes MI depletion and oxidative injury in cervical cancer cells

**Figure 6:**
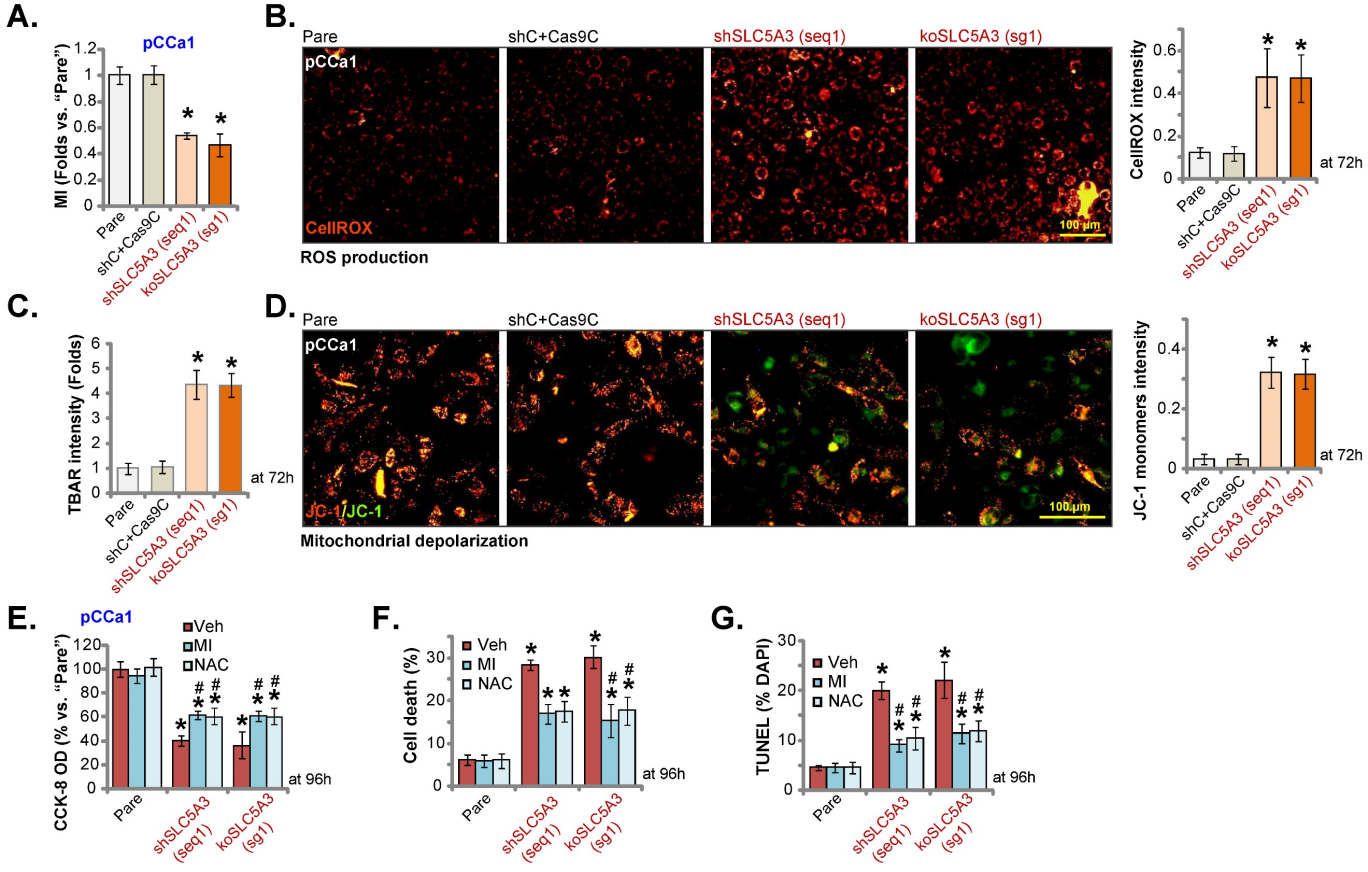
***SLC5A3* knockdown/KO promotes MI depletion and oxidative injury in cervical cancer cells.** pCCa-1 cells with described genetic treatments were cultured; Myo-inositol (MI) contents were examined (**A**). Cellular ROS contents were tested through quantifying the CellROX red fluorescence (**B**); TBAR intensity was measured (**C**); Mitochondrial depolarization was examined by quantifying the JC-1 green monomers (**D**). The above cells were also co-treated with MI (2.5 mM), NAC (400 μM) or the vehicle (“Veh”) control for 96h, cell viability (CCK-8,**E**), death (**F**) and apoptosis (TUNEL assays, **G**) were tested. * ***P*** < 0.05 *vs.* “Pare”/“shC” cells. **^#^ *P*** < 0.05 vs. “Veh” treatment (**E**-**G**).

SLC5A3 is involved in transporting MI 16, 29, 30. The cellular MI contents were downregulated in sh-SLC5A3 (seq1)-transduced pCCa-1 cells (Figure **6A**). Consistently, transduction with the koSLC5A3 (sg1) construct (Figure **5**) downregulated the cellular MI contents (Figure **6A**). SLC5A3 exerts anti-oxidant effects and downregulates reactive oxygen species (ROS) production in neuronal cells 31. In this study, SLC5A3 knockdown or KO upregulated oxidative stress in cervical cancer cells. The CellROX dye intensity was upregulated in pCCa-1 cervical cancer cells transduced with sh-SLC5A3 (seq1) or koSLC5A3 (sg1) (Figure **6B**). The thiobarbituric acid reactive substance (TBAR) values were upregulated in *SLC5A3* knockdown or KO pCCa-1 cells, suggesting the upregulation of lipid peroxidation (Figure **6C**). JC-1 monomer formation further supported the depolarization of mitochondria in SLC5A3-depleted pCCa-1 cells (Figure **6D**). Transduction with shC and Cas9C (“shC+Cas9C”) did not affect MI contents (Figure **6A**) and ROS production/oxidative injury (Figure **6B**-**D**) in pCCa-1 cells.

To investigate the contribution of MI downregulation and oxidative injury to the anti-cervical cancer effects of SLC5A3 depletion, cells were exogenously treated with MI or the anti-oxidant N-acetyl cysteine (NAC). Treatment with MI (2.5 mM) or NAC (400 μM) significantly mitigated the *SLC5A3* knockdown/KO-induced downregulation of pCCa-1 cell viability (Figure **6E**) and upregulation of pCCa-1 cell death and apoptosis (Figure **6F**-**G**). However, treatment with MI or NAC alone did not decrease pCCa-1 cell viability and induce death/apoptosis (Figure **6E**-**G**). These results indicate that *SLC5A3* depletion promotes cervical cancer cell death by downregulating MI and ROS production.

### *SLC5A3* knockdown/KO inhibits Akt-mTOR activation

**Figure 7:**
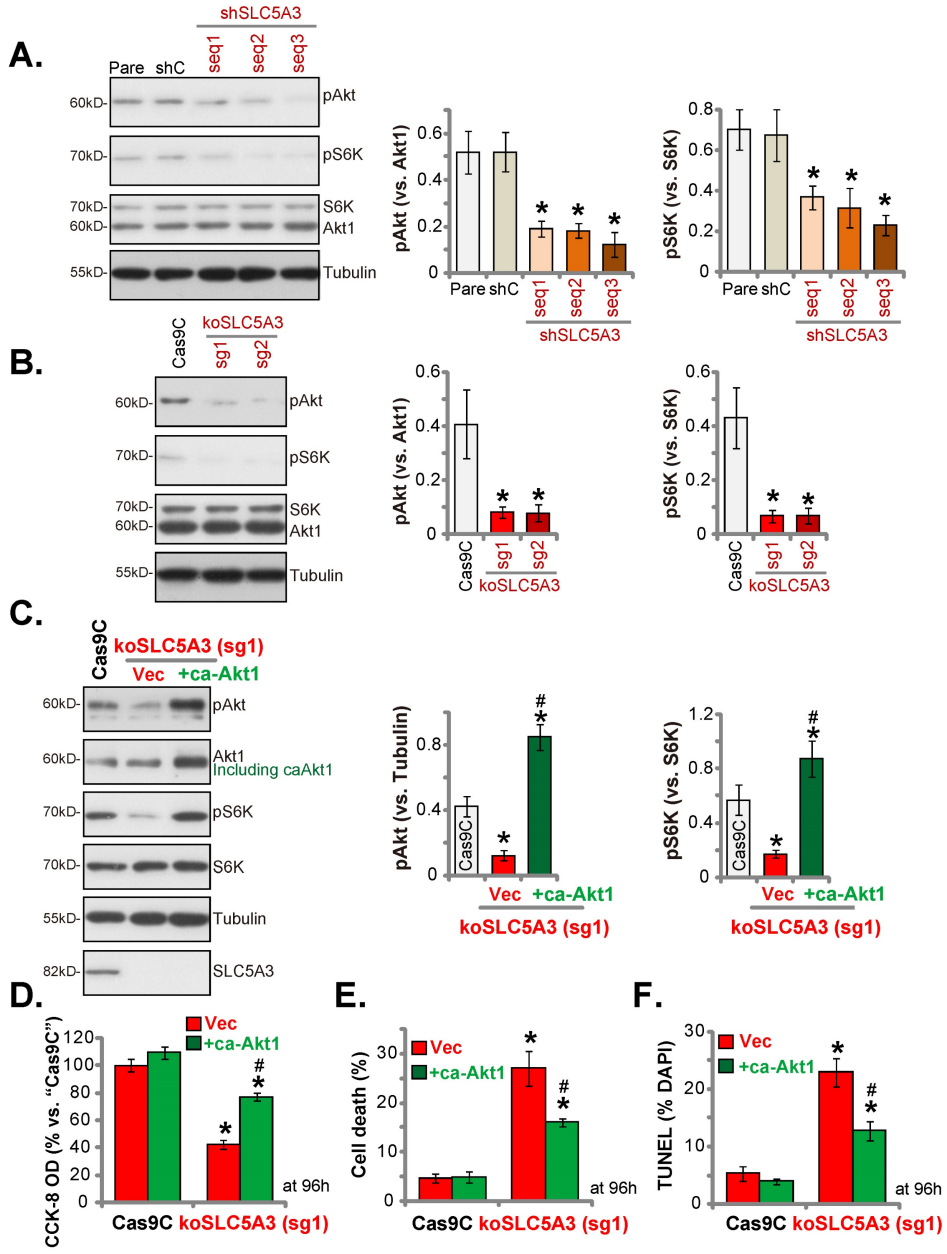
**SLC5A3 silencing/KO inhibits Akt-mTOR activation.** pCCa-1 cells with the applied lentiviral SLC5A3 shRNA [shSLC5A3 (seq1/2/3)], control shC, the CRISPR/Cas9-SLC5A3-knockout (KO) construct encoded two different sgRNAs: koSLC5A3 (sg1) and koSLC5A3 (sg2), or Cas9C were cultured, listed proteins were shown (**A** and **B**). The koSLC5A3 (sg1) pCCa-1 cells were further stably transduced with the S473D caAkt1 or vector (“Vec”), listed proteins were examined (**C**); After culture of 96h, cell viability (**D**), death (**E**) and cell apoptosis (TUNEL assays, **F**) were examined. * ***P*** < 0.05 *vs.* “Pare”/“Cas9C” cells. **^#^ *P*** < 0.05 *vs.* “Vec” cells (**C**-**F**).

Inositol second messengers can actively regulate multiple signaling cascades involved in cancer progression. In particular, inositols are reported to activate the Akt cascade 32. Akt-mTOR cascade hyperactivation is important for cervical cancer growth 33, 34. Transduction with sh-SLC5A3 (seq1/2/3) markedly downregulated the phosphorylation of Akt1 (Ser-473) and S6K (mTOR marker protein) (Figure **7A**), resulting in Akt-mTOR cascade suppression. Similarly, transduction with koSLC5A3 (sg1) markedly downregulated the phosphorylation of Akt1 and S6K (Figure **7B**). To activate the Akt-mTOR cascade, an S473D constitutively active Akt1 (“caAkt1”) construct was stably transduced into koSLC5A3 (sg1)-transduced pCCa-1 cells, which upregulated the phosphorylation of Akt1 and S6K (Figure **7C**). Transduction with caAkt1 suppressed the SLC5A3 KO-induced downregulation of pCCa-1 cell viability (Figure **7D**) and upregulation of cell death (Figure **7E**) and apoptosis (Figure **7F**).

### Ectopic *SLC5A3* overexpression exerts pro-cervical cancer effects

**Figure 8:**
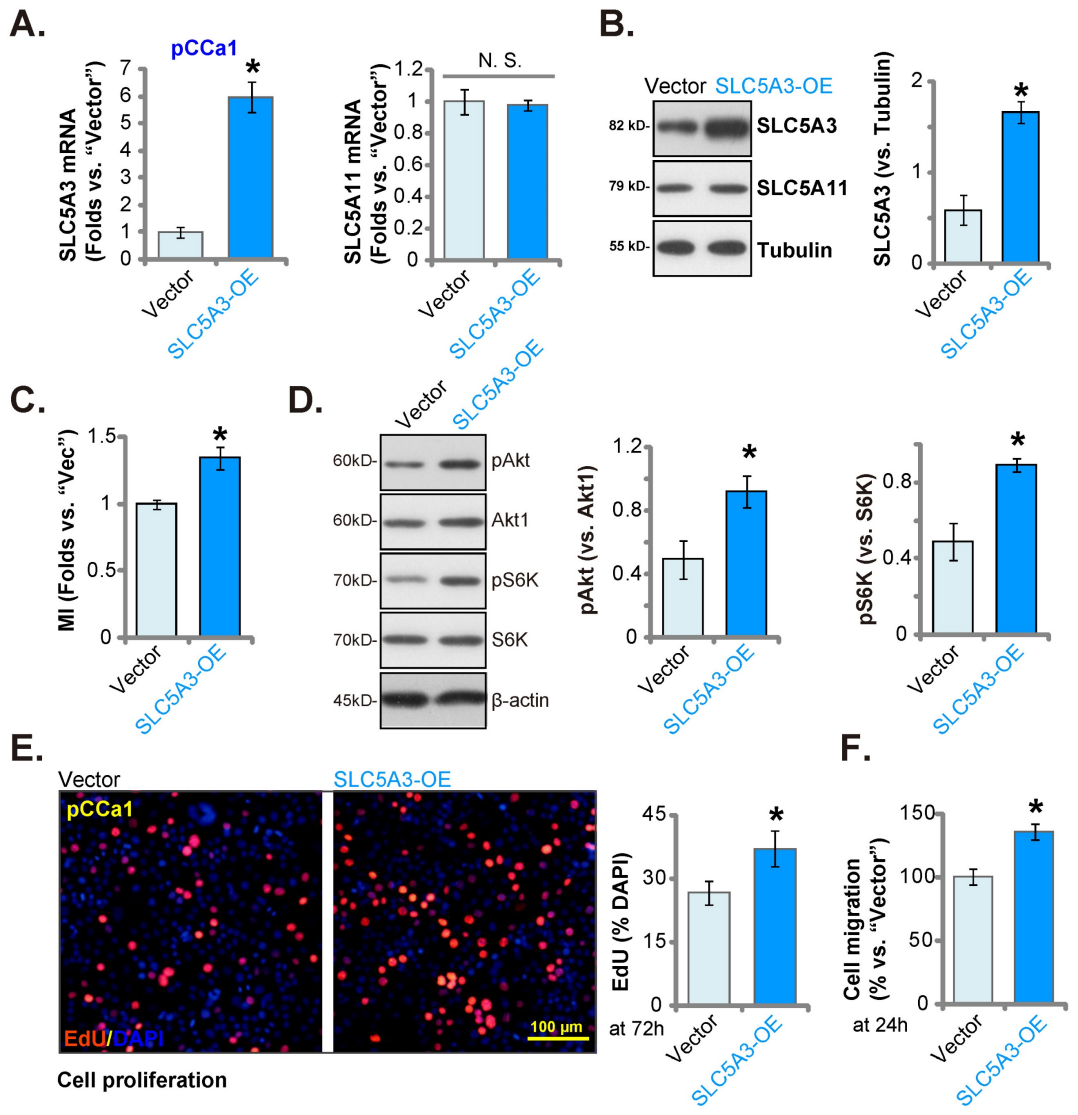
** Ectopic SLC5A3 overexpression exerts pro-cervical cancer effects.** SLC5A3-overexpressing pCCa-1 cells (“SLC5A3-OE”) or empty vector (“Vector”) were established, the listed mRNAs and proteins were measured (**A**, **B** and **D**); Myo-inositol (MI) contents were measured (**C**). The exact same number of the pCCa-1 cells were cultured for designated hours, EdU incorporation (**E**) and migration (**F**) were examined. * ***P*** < 0.05 *vs.* “Vector” cells.

Next, a lentiviral SLC5A3 overexpression construct (encoding *SLC5A3* cDNA) was stably transduced into pCCa-1 cells (“SLC5A3-OE” cells). The *SLC5A3* mRNA levels were upregulated by 5-6 fold in SLC5A3-OE cells (Figure **8A**) but the *SLC5A11* mRNA levels were unaltered (Figure **8A**). Consistently, the SLC5A3 protein levels were upregulated in SLC5A3-OE cells (Figure **8B**) but the SLC5A11 protein levels were unaffected (Figure **8B**). The cellular MI contents were upregulated in SLC5A3-OE cells (Figure **8C**). Additionally, SLC5A3 overexpression upregulated Akt-mTOR cascade activation and promoted the phosphorylation of Akt1 and S6K in pCCa-1 cells (Figure **8D**). Furthermore, ectopic SLC5A3 overexpression increased EdU incorporation, suggesting that SLC5A3 overexpression promoted pCCa-1 cell proliferation (Figure **8E**). The *in vitro* migration of SLC5A3-OE cells was also strengthened (Figure **8F**). These findings indicate that ectopic SLC5A3 overexpression enhanced cervical cancer cell proliferation and migration.

### TonEBP-*SLC5A3* promoter binding is upregulated in cervical cancer

**Figure 9:**
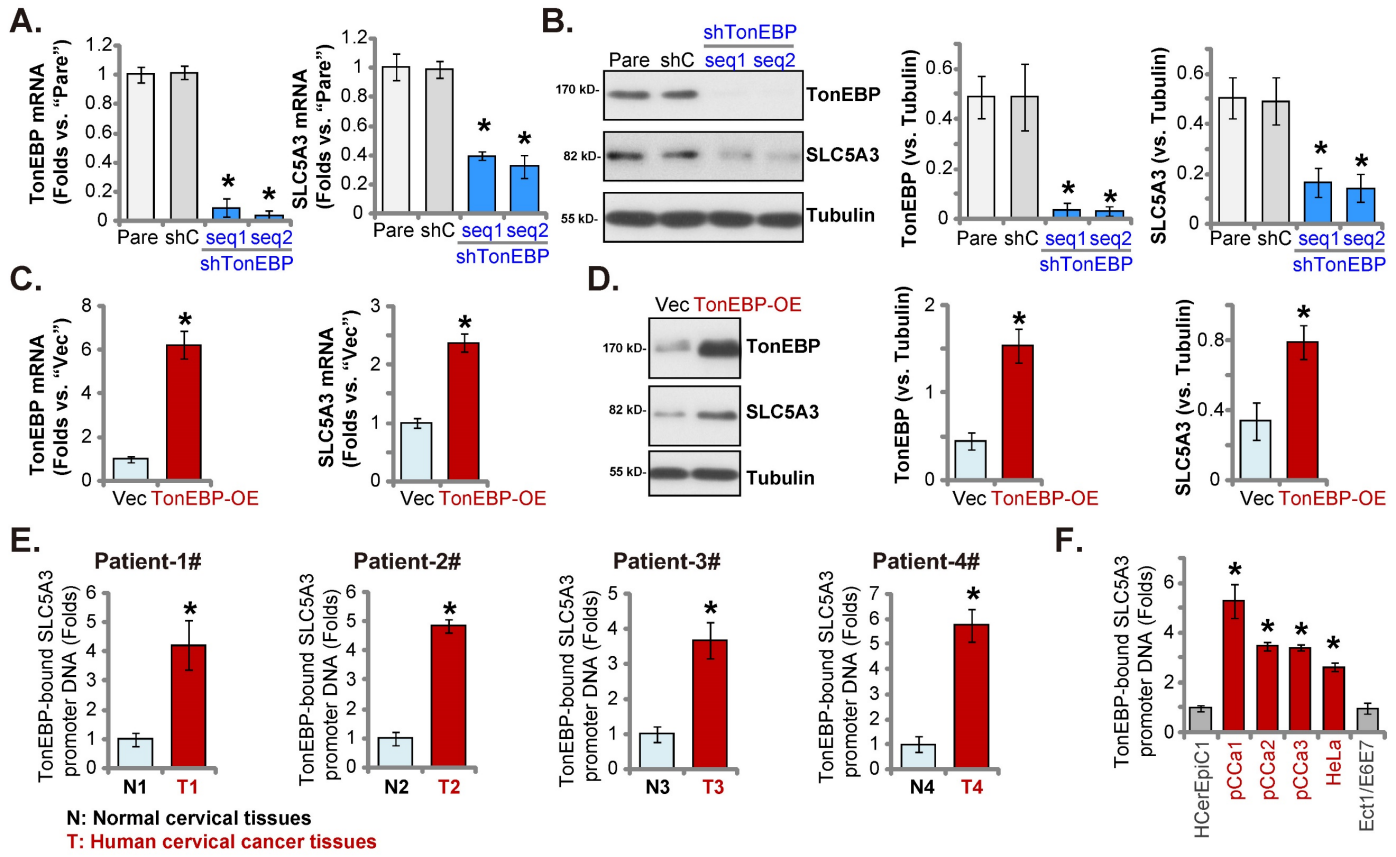
**TonEBP-*SLC5A3* promoter binding is upregulated in cervical cancer.** pCCa-1 cells with the applied lentiviral TonEBP shRNA (shTonEBP-seq1/shTonEBP-seq2, with two different sequences), shC, TonEBP-overexpressing construct (“TonEBP-OE”) or control vector ( “Vec”) were formed, with listed mRNAs/proteins examined (**A**-**D**). ChIP assay results revealed the relative level of the *SLC5A3* promoter DNA-bound to TonEBP in listed tissues (**E**) and cells (**F**). * ***P*** < 0.05 *vs.* “shC”/“Vec”/“N”/“HCerEpiC1”.

The upregulated *SLC5A3* mRNA and protein levels in cervical cancer contribute to cancer cell growth. Next, the potential mechanism of SLC5A3 upregulation was examined by focusing on the transcriptional mechanism. Johnson *et al.* demonstrated that TonEBP (NFAT5) is an important transcription factor of SLC5A3 and that *TonEBP* expression was correlated with *SLC5A3* expression 35. *TonEBP* (*NFAT5*) was the second top differentially expressed gene (DEG) that was positively correlated with SLC5A3 expression in TCGA cervical cancer cohort (Figure **1F-G**). The pCCa-1 primary cells were transduced with lentiviral particles encoding TonEBP shRNAs (sh-TonEBP-seq1 and sh-TonEBP-seq2). Stable *TonEBP* knockdown cells were obtained after selection. Transduction with TonEBP shRNAs markedly downregulated the expression of TonEBP (Figure **9A**-**B**) and SLC5A3 (Figure **9A**-**B**) in pCCa-1 cells.

*TonEBP* cDNA sequence-encoding lentiviral particles were transduced into pCCa-1 cells to obtain stable TonEBP-overexpressing cells (“*TonEBP*-OE” cells). The* TonEBP* Mrna and protein levels, as well as the *SLC5A3* mRNA and protein levels, were upregulated in TonEBP-OE pCCa-1 cells (Figure **9C**-**D**). These results indicate that TonEBP is a key transcription factor of *SLC5A3* in cervical cancer cells.

The results of ChIP assay (Figure **9E**) revealed that the binding of TonEBP to the proposed *SLC5A3* promoter 35 was significantly upregulated in the cervical cancer tissues of four patients but was relatively low in matched adjacent non-cancerous cervical epithelial tissues (Figure **9E**). The binding of TonEBP to the *SLC5A3* promoter was strong in pCCa-1/pCCa-2/pCCa-3 primary cells and immortalized HeLa cells (Figure **9F**) but weak in HCerEpC1 and Ect1/E6E7 cells (Figure **9F**). Therefore TonEBP-*SLC5A3* promoter DNA binding increasing in cervical cancer is key for SLC5A3 upregulation.

### *SLC5A3* knockdown arrests the growth of cervical cancer xenografts

**Figure 10:**
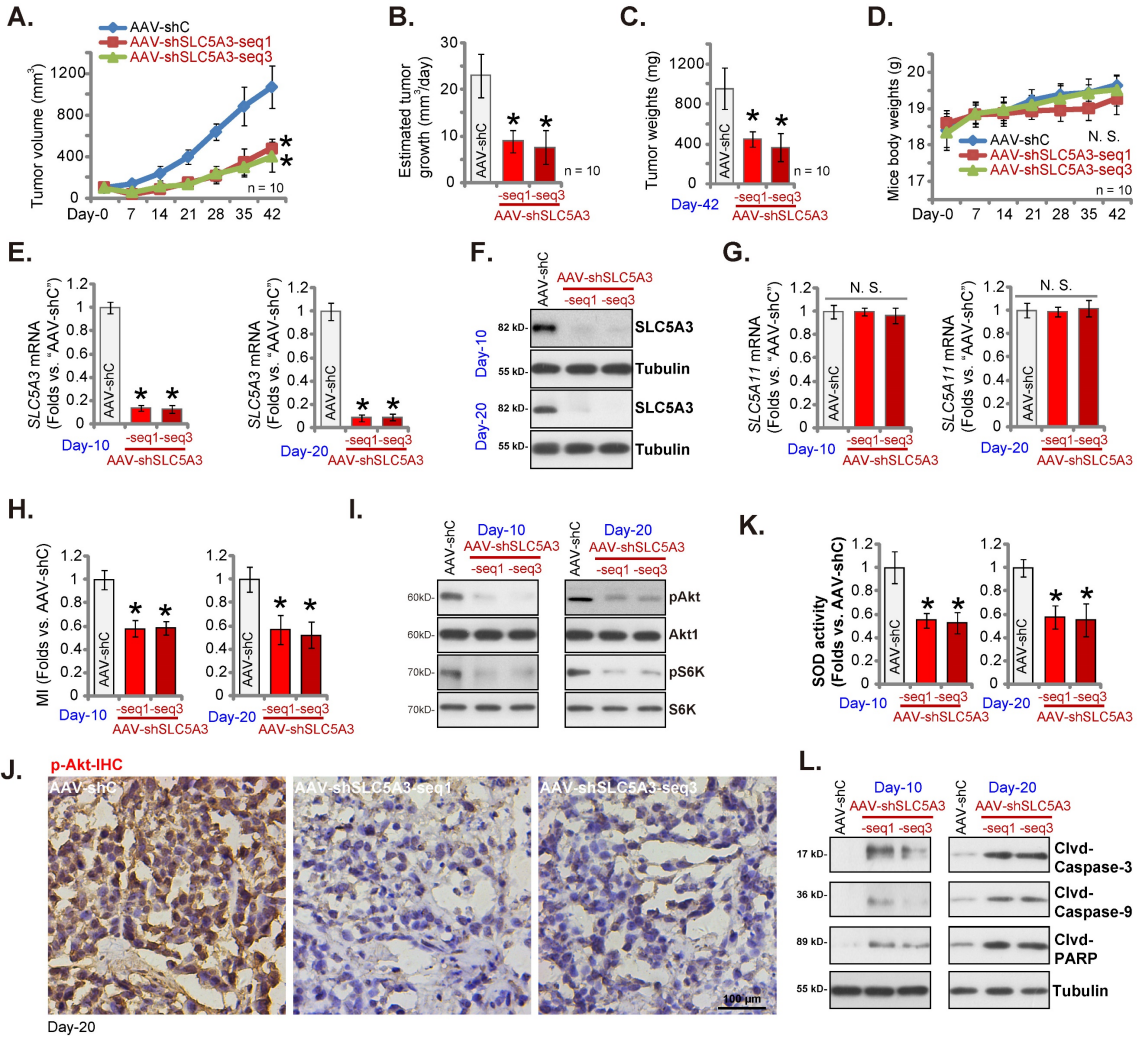
***SLC5A3* knockdown arrests the growth of cervical cancer xenografts.** The nude mice bearing pCCa-1 xenografts were intratumorally injected with SLC5A3 shRNA-expressing AAV [“AAV-shSLC5A3 (seq1)/AAV-shSLC5A3 (seq3)”] or the shC-expressing AAV (“AAV-shC”), every 48h for 10 days; Weekly pCCa-1 xenograft volumes (**A**) and animal weights (**D**) were measured; Daily pCCa-1 xenograft growth was estimated (**B**). All pCCa-1 xenografts were isolated at Day-42 and tumor weights recorded (**C**). In the described pCCa-1 xenograft tissues, expression of listed mRNAs/proteins (**E**-**G**, **I** and **L**), myo-inositol (MI) contents (**H**) and SOD activity (**K**) were measured; The pCCa-1 xenograft tissue slides were subject to IHC staining of p-Akt (**J**). *P < 0.05 versus “AAV-shC” group.

Nude mice were subcutaneously injected with pCCa-1 primary cells, resulting in the formation of xenograft tumors after three weeks (day 0). The SLC5A3 shRNA-encoding adeno-associated virus (AAV) constructs [AAV-sh-SLC5A3 (seq1) and AAV-sh-SLC5A3 (seq3)] were intratumorally injected into mice. Mice in the control group were intratumorally injected with shC-encoding AAV (“AAV-shC”) construct. The AAV constructs were injected once every two days five times. As shown in Figure **10A**, the weekly xenograft growth curve revealed that AAV-sh-SLC5A3 injection impaired pCCa-1 xenograft growth. The volume of AAV-sh-SLC5A3 (seq1/3)-injected tumors was lower than that of the AAV-shC-injected tumors (Figure **10A**). Moreover, AAV-sh-SLC5A3 injection suppressed daily pCCa-1 xenograft growth (estimated in mm^3^ per day) (Figure **10B**). All pCCa-1 xenografts were carefully isolated on day 42. The weight of AAV-sh-SLC5A3-injected pCCa-1 xenografts significantly decreased (Figure **10C**). The mean bodyweight was not significantly different between the three groups (Figure **10D**).

Next, the signaling cascades in the AAV-sh-SLC5A3-injected tumors were analyzed. On days 10 and 20, one pCCa-1 xenograft was isolated from each group. The *SLC5A3* mRNA (Figure **10E**) and protein (Figure **10F**) levels were markedly downregulated in AAV-sh-SLC5A3 (seq1/3)-injected tumors but the *SLC5A11* mRNA levels were unchanged (Figure **10G**). Additionally, the MI contents (Figure **10H**) and the phosphorylation of Akt1 and S6K (Figure **10I**) were downregulated in AAV-sh-SLC5A3 (seq1/3)-injected pCCa-1 xenograft tissues. IHC analysis further confirmed the suppression of Akt1 phosphorylation in AAV-sh-SLC5A3 (seq1/3)-injected pCCa-1 xenografts (Figure **10J**).

The superoxide dismutase (SOD) activity was significantly downregulated (Figure **10K**) in AAV-sh-SLC5A3 (seq1/3)-injected pCCa-1 xenograft tissues, indicating the induction of oxidative injury. The levels of the cleaved apoptosis marker proteins were upregulated in AAV-sh-SLC5A3 (seq1/3)-injected pCCa-1 xenograft tissues (Figure **10L**). Thus, *SLC5A3* knockdown downregulated MI contents, suppressed Akt-mTOR cascade activation, promoted oxidative injury, and induced apoptosis in pCCa-1 xenografts.

### *SLC5A3* KO hinders pCCa-1 cervical cancer xenograft growth

**Figure 11:**
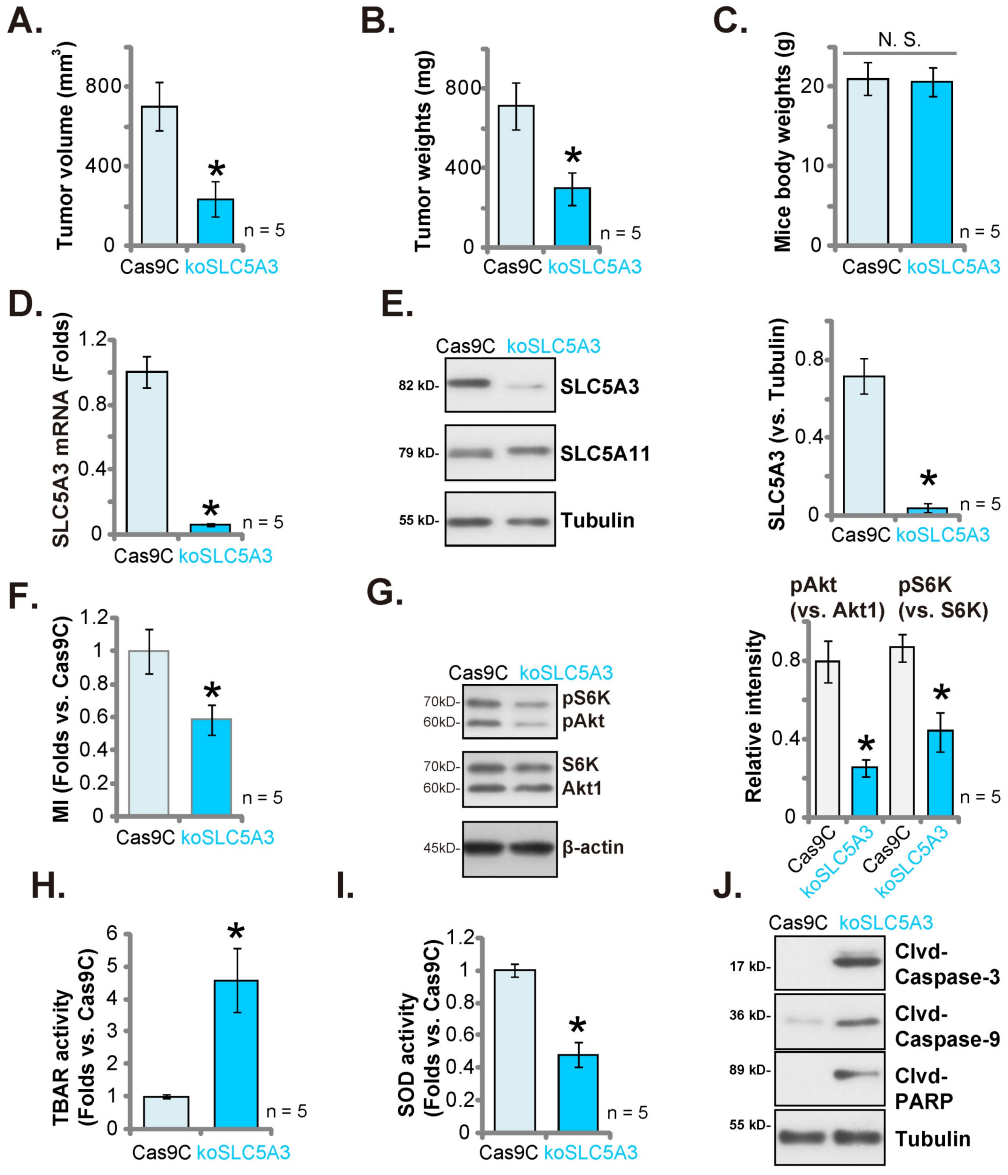
***SLC5A3* KO suppresses cervical cancer xenograft growth.** The koSLC5A3 pCCa-1 cells (with sg1) orCas9C control were *s.c.* injected to the nude mice, after 60 days pCCa-1 xenograft volumes (**A**) and weights (**B**) as well as animal body weights (**C**) were recorded. In the fresh pCCa-1 xenograft tissues listed mRNAs/proteins were measured (**D**, **E**, **G** and **J**); Myo-inositol (MI) contents (**F**), TBAR intensity (**H**) and SOD activity (**I**) were tested. *P < 0.05 versus “Cas9C” group.

Next the primary pCCa-1 cells with the CRISPR/Cas9-SLC5A3-knockout (KO) construct (“koSLC5A3”, with sg1, see Figure **5**) or Cas9C (see Figure **5**) were *s.c.* injected to nude mice. Sixty days later, the pCCa-1 xenografts were isolated. The sizes and weights of koSLC5A3 (sg1)-injected pCCa-1 xenografts were markedly downregulated (Figure **11A**-**B**) when compared with those of Cas9C pCCa-1 xenografts. The bodyweight was not significantly different between the groups (Figure **11C**). Tumor lysates were prepared from koSLC5A3 (sg1)-injected and Cas9C-injected pCCa-1 xenografts. The SLC5A3 expression levels were downregulated in the lysates of koSLC5A3 (sg1)-injected xenograft tissues (Figure **11D**-**E**) but the SLC5A11 protein levels were unaffected (Figure **11E**). Additionally, the MI contents (Figure **11F**) and the phosphorylation of Akt1 and S6K (Figure **11G**) were downregulated in koSLC5A3 (sg1)-injected pCCa-1 xenograft tissues. Furthermore, the TBAR values were upregulated in koSLC5A3 (sg1)-injected pCCa-1 xenograft tissues (Figure **11H**), indicating upregulation of lipid peroxidation. The SOD activity was downregulated in koSLC5A3 (sg1)-injected xenograft tissues, indicating upregulation of oxidative injury (Figure **11I**). The levels of the cleaved apoptosis marker proteins were upregulated in koSLC5A3 (sg1)-injected xenograft tissues, suggesting apoptosis activation (Figure **11J**). Thus, SLC5A3 KO significantly impaired pCCa-1 cervical cancer xenograft growth.

### TonEBP silencing inhibits pCCa-1 cervical cancer xenograft growth

**Figure 12:**
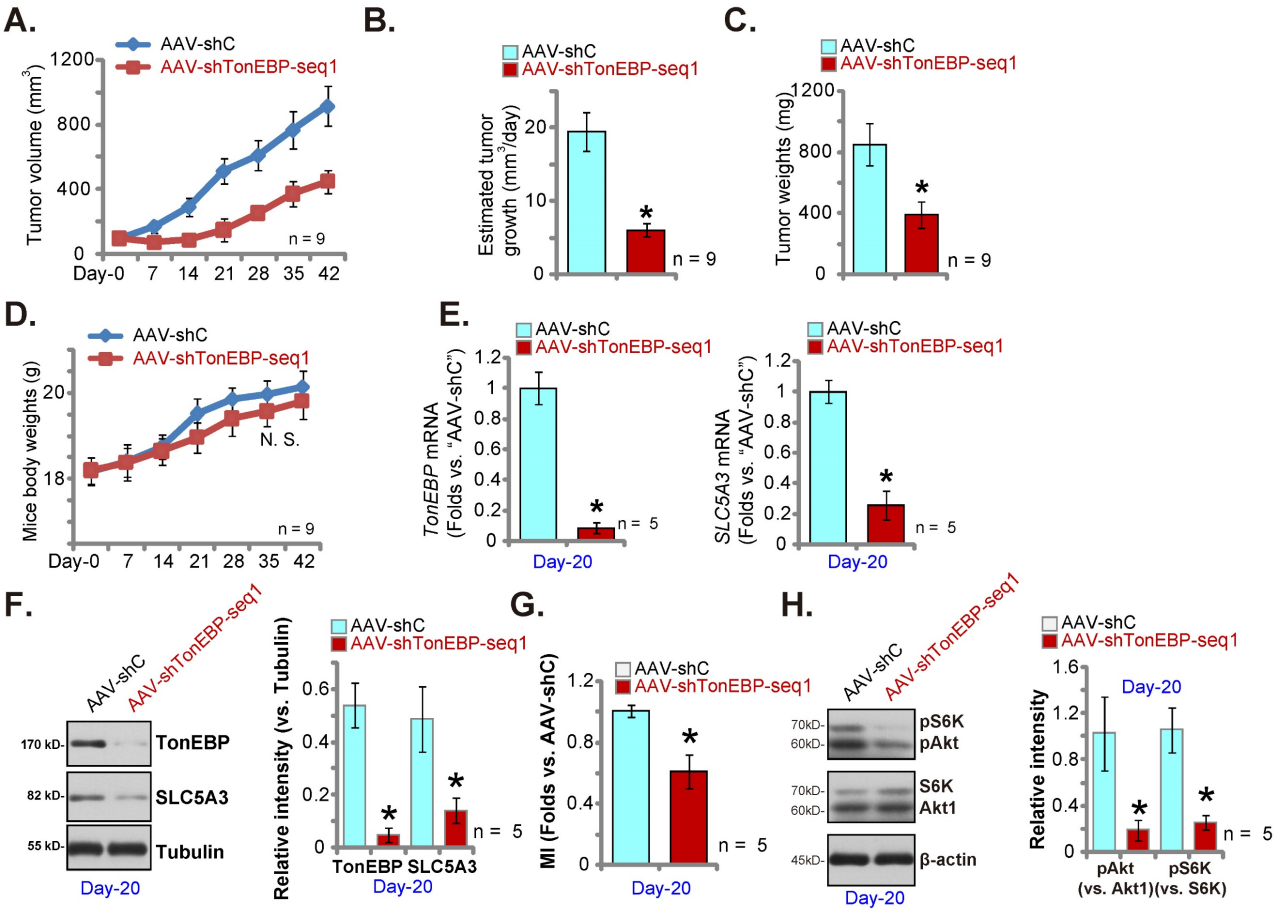
** TonEBP silencing inhibits pCCa-1 cervical cancer xenograft growth.** The nude mice bearing pCCa-1 xenografts were intratumorally injected with TonEBP shRNA-expressing AAV (“AAV-shTonEBP-seq1”) or AAV-shC, every 48h for 10 days; Weekly pCCa-1 xenograft volumes (**A**) and body weights of the mice (**D**) were recorded; Daily pCCa-1 xenograft growth was estimated (**B**). All pCCa-1 xenografts were isolated at Day-42 and xenograft weights were recorded (**C**). At Day-20, one pCCa-1 xenograft per group was isolated; Listed mRNAs/proteins (**E**, **F** and **H**) and myo-inositol (MI) contents (**G**) were tested. *P < 0.05 versus “AAV-shC” group.

We further hypothesized that silencing of the transcription factor TonEBP should downregulate SLC5A3 expression and inhibit cervical cancer cell *in vivo* growth. Therefore, TonEBP shRNA AAV (“AAV-shTonEBP-seq1”) were intratumorally injected to mice bearing pCCa-1 tumors. The control group mice received AAV-shC (see Figure **10**). AAV-shTonEBP-seq1 hindered pCCa-1 xenograft growth *in vivo* (Figure **12A**). The daily pCCa-1 xenograft growth was significantly decreased after AAV-shTonEBP-seq1 injection (Figure **12B**). Analyzing the isolated pCCa-1 xenografts at Day-42 found that AAV-shTonEBP-seq1-treated pCCa-1 xenografts were lighter than AAV-shC xenografts (Figure **12C**), with no difference in animal weights detected (Figure **12D**).

At Day-20, one pCCa-1 xenograft per group was isolated. *TonEBP* and *SLC5A3* mRNA levels were both decreased in AAV-shTonEBP-seq1-treated pCCa-1 xenograft tissues (Figure **12E**). TonEBP and SLC5A3 protein expression was decreased as well (Figure **12F**). As a consequence, MI contents (Figure **12G**) and Akt-S6K phosphorylation (Figure **12H**) were reduced in TonEBP-silenced pCCa-1 xenograft tissues. These results together supported that silencing the transcription factor TonEBP downregulated SLC5A3 and inhibited pCCa-1 cervical cancer xenograft growth.

## Discussion

Human papillomavirus (HPV) vaccines, cervical cancer screening, and advances in diagnosis and adjuvant treatments have contributed to the decreased incidence of cervical cancer, especially in developed countries 3, 36, 37. Currently, cervical cancer does not feature among the top 10 malignant tumors in the United States. However, in China and other developing countries, cervical cancer is the second most common tumor among women and is associated with high cancer-related mortality rates each year 3, 36, 37.

We hypothesized that *SLC5A3* is an important oncogenic gene for cervical cancer. Bioinformatics analysis revealed that *SLC5A3* is upregulated in cervical cancer tissues. The upregulated *SLC5A3* expression was correlated with both poor survival and poor PFI. SLC5A3 upregulation was confirmed in clinical cancer tissues and different cervical cancer cells. *SLC5A3* knockdown or KO suppressed cervical cancer cell viability, proliferation, and migration and induced cell death and apoptosis. In contrast, SLC5A3 overexpression promoted cancer cell proliferation and migration. Transduction with sh-SLC5A3 AAV constructs suppressed pCCa-1xenograft growth in nude mice. Similarly, SLC5A3 KO also inhibited pCCa-1 cervical cancer xenograft growth.

MI is used to treat reproductive and metabolic disorders 38. Previous studies have demonstrated that MI decreases body mass index and increases insulin sensitivity in female patients with polycystic ovary syndrome 39. Additionally, MI regulates steroidogenesis and modulates androgen and estrogen contents 39. Lin *et al.* demonstrated that SLC5A3 is a key metabolic factor for AML using an *in vivo* CRISPR screening platform 40. SLC5A3 is critical for AML cell proliferation. CRISPR-mediated *SLC5A3* KO markedly suppressed AML orthotopic xenograft growth *in vivo* and induced apoptosis 40. SLC5A3-induced MI import promotes AML cell proliferation 40. Wei *et al.* demonstrated that MI promotes nutrient dependency in AML and that MI imported via SLC5A3 maintained AML cell proliferation 16. This study demonstrated that cellular MI contents were upregulated in *SLC5A3* knockdown/KO cervical cancer cells but were upregulated in SLC5A3-overexpressing cells. MI was also downregulated in sh-SLC5A3 AAV-injected or koSLC5A3 (sg1)-injected cervical cancer xenograft tissues. MI supplementation alleviated SLC5A3 KO-induced apoptosis and cell death in cervical cancer cells. Thus, SLC5A3-dependent MI import is critical for cervical cancer cell growth.

Akt-mTOR cascade hyperactivation is a driving factor for cervical cancer growth and progression 33, 34, 41. Various pharmacological inhibitors or monoclonal antibodies against the Akt-mTOR cascade-related factors exerted therapeutic effects on cervical cancer 33, 34, 41. This study proposed that SLC5A3 is essential for Akt-mTOR signaling activation. *SLC5A3* knockdown/KO significantly decreased the phosphorylation of Akt1 and S6K in primary cervical cancer cells. The Akt-mTOR cascade was inhibited in sh-SLC5A3 AAV-injected cervical cancer xenograft tissues and SLC5A3 KO xenograft tissues. In contrast, SLC5A3 overexpression upregulated the phosphorylation of Akt1 and S6K. The reactivation of Akt-mTOR by caAkt1 suppressed SLC5A3 KO-induced cervical cancer cell death. Thus, SLC5A3 mediates cervical cancer cell growth, at least partially, by promoting Akt-mTOR activation.

SLC5A3, which is widely expressed in different human tissues, is important for cellular osmoregulation 11. Previous studies have reported that SLC5A3 regulates inflammatory cell infiltration during the progression of sporadic inclusion body myositis 29. Zhou *et al.* revealed the potential anti-oxidant activity of SLC5A3 and demonstrated that *SLC5A3* knockdown augmented oxidative stress in human neuroblastoma cells 31. Long non-coding RNA NORAD upregulated SLC5A3 expression by sponging microRNA-204-5p and consequently alleviates oxidative injury and suppressed cell death in neuroblastoma cells 31. The ROS and oxidative stress levels were upregulated in *SLC5A3* knockdown cervical cancer cells and SLC5A3 knockdown/KO xenograft tissues. The anti-oxidant NAC suppressed SLC5A3 KO-induced cytotoxicity against cervical cancer cells. Thus, SLC5A3 depletion exerts anti-cervical cancer effects by inducing oxidative stress.

This study demonstrated that TonEBP is the key transcription factor for *SLC5A3* in cervical cancer. *TonEBP* was the second top DEG that was positively correlated with *SLC5A3* in TCGA cohort. The *SLC5A3* mRNA and protein expression levels were downregulated upon *TonEBP* knockdown but were upregulated upon ectopic *TonEBP* overexpression in cervical cancer cells. The binding of TonEBP to the *SLC5A3* promoter was upregulated in various cervical cancer tissues/cells and may contribute to SLC5A3 upregulation. *TonEBP* knockdown suppressed SLC5A3 expression and inhibited pCCa-1 tumor growth *in vivo*. Thus, TonEBP upregulates SLC5A3 expression and promotes cervical cancer cell growth.

## Conclusion

The upregulated SLC5A3 promotes cervical cancer cell growth.

## Supplementary Material

Supplementary figure data.
