# Supplementary material for: SLC5A3 is important for cervical cancer cell growth
Source: Int J Biol Sci. 2023 May 27;19(9):2787–802. doi: 10.7150/ijbs.84570 (PMC10266070; doi:10.7150/ijbs.84570)

Figure S1: The uncropped blotting images.

Figure 2.

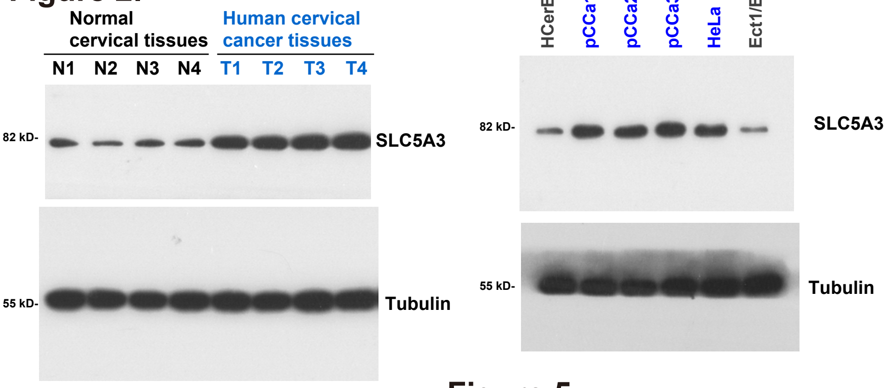

Figure 3.

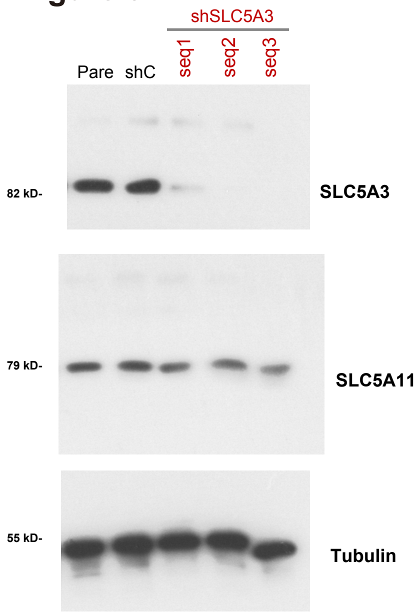

Figure 4.

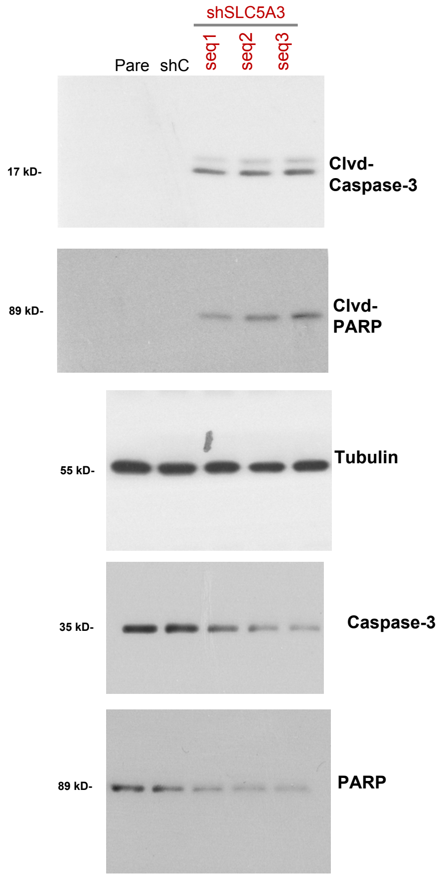

Figure 5.

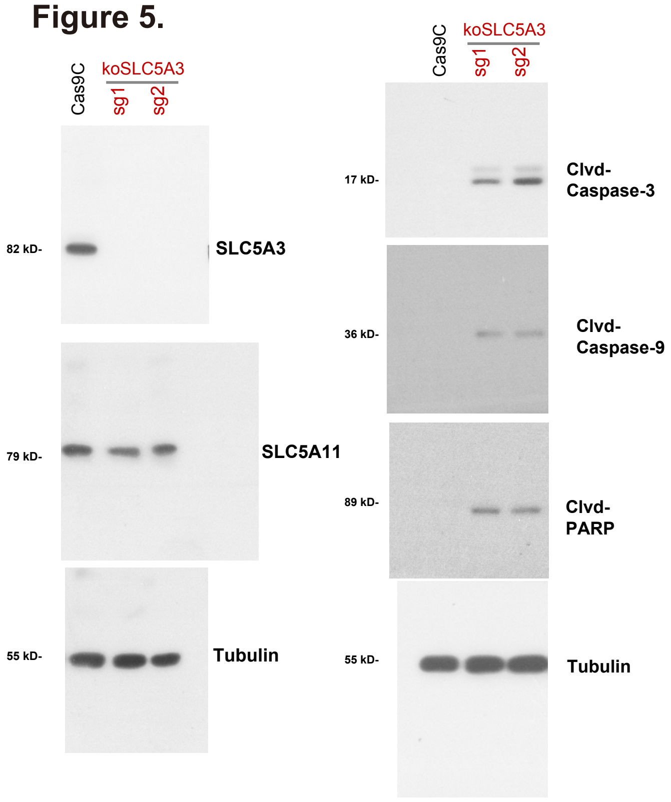

Figure 7.

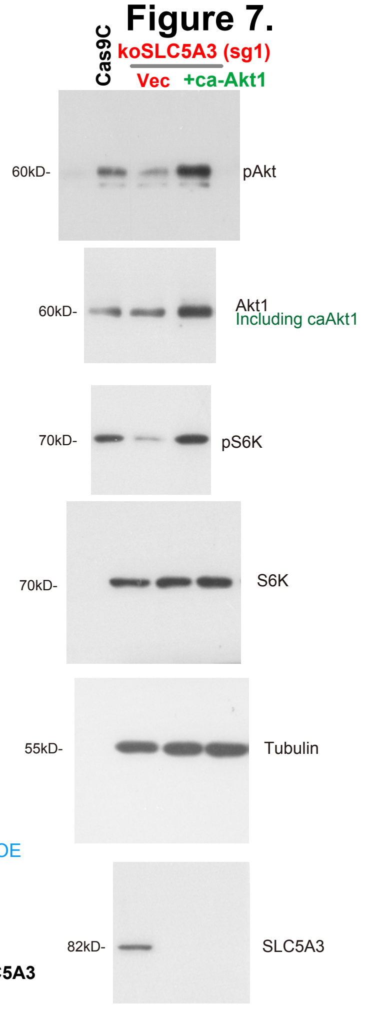

Figure 8.

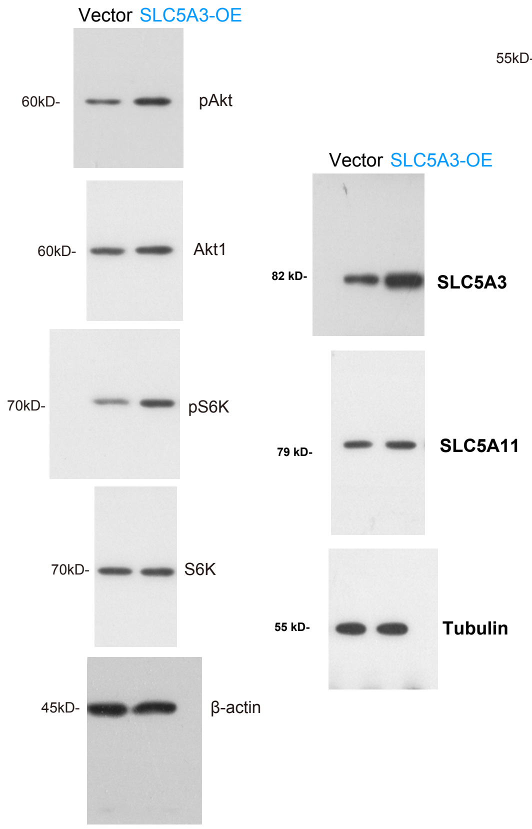

Figure 7.

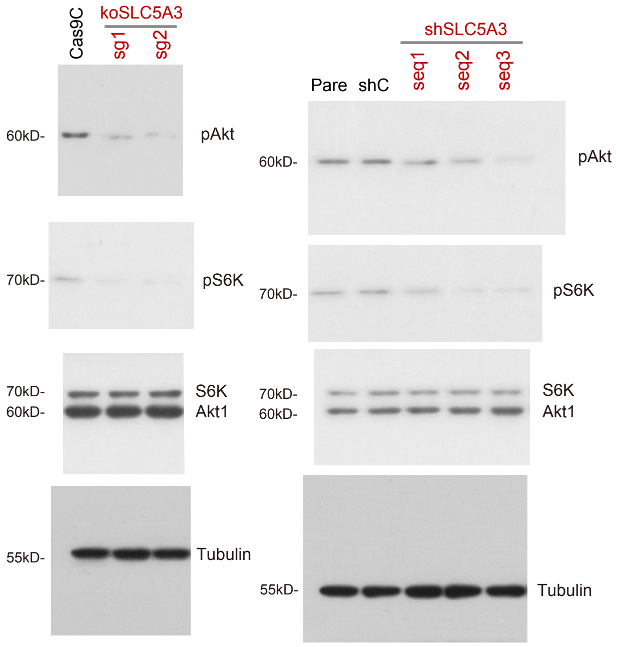

Figure 9.

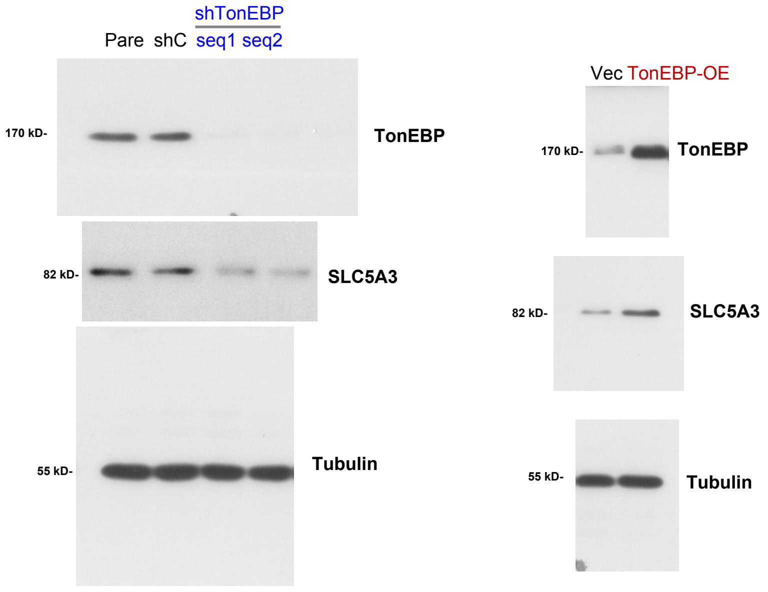

Figure 11.

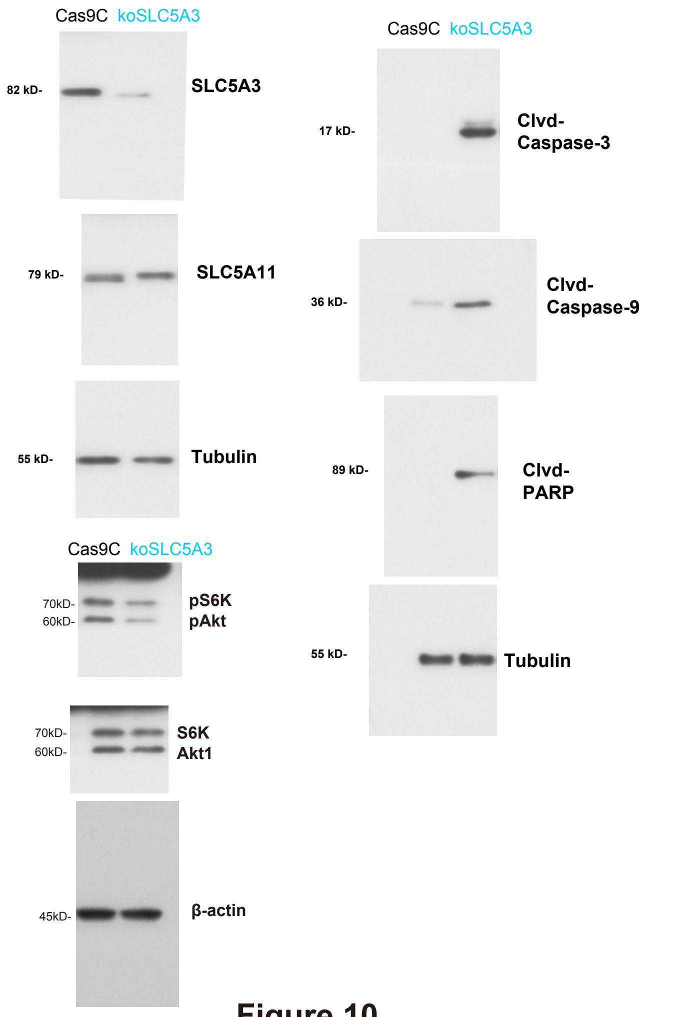

Figure 10.

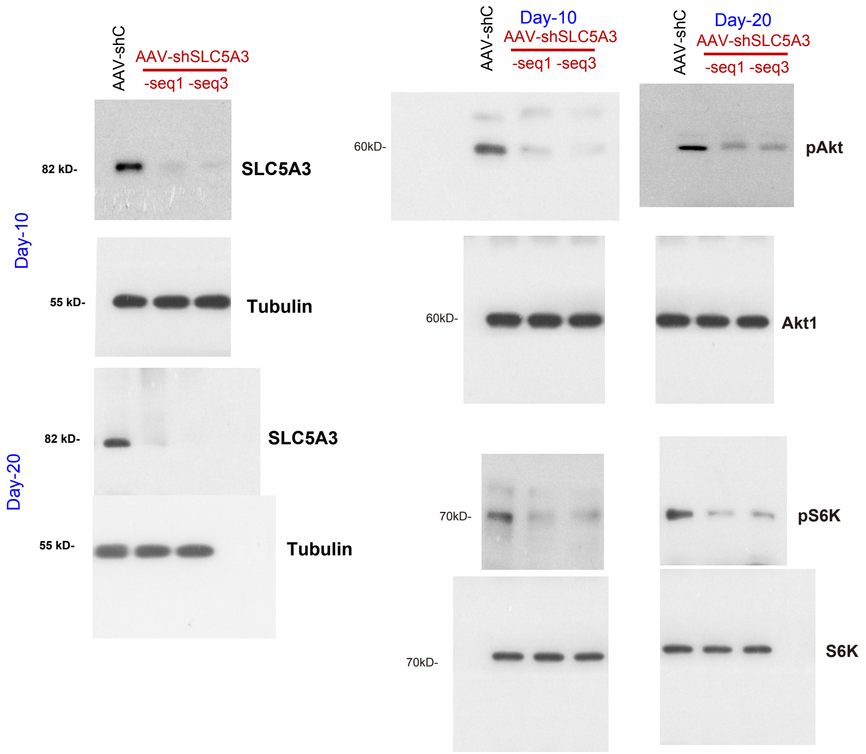

Figure 10.

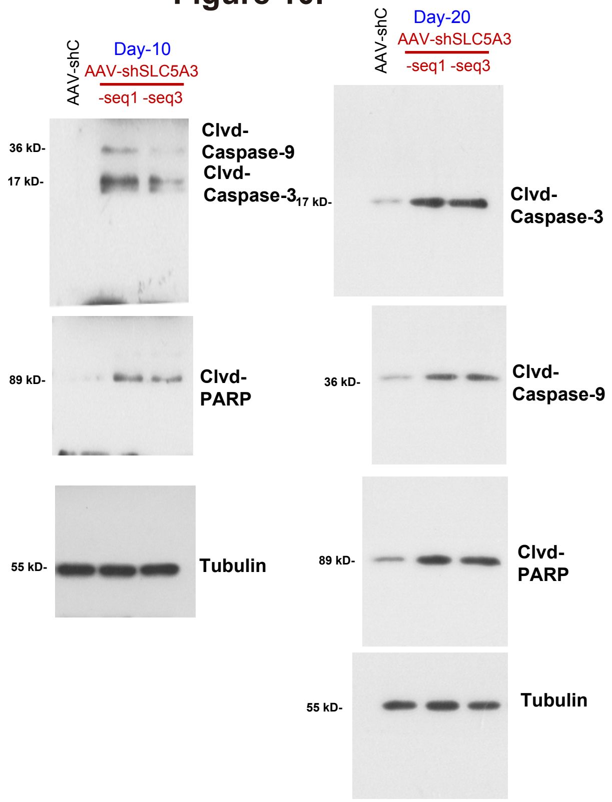

Figure 12.

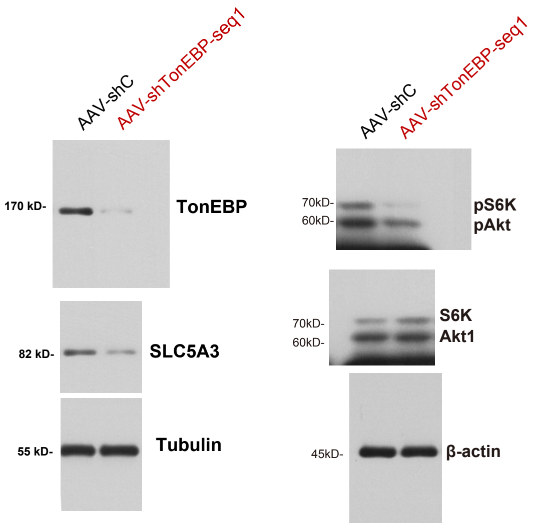

Supplement: Supplementary file 1 — Supplementary figure data. [file ijbsv19p2787s1.pdf]
